# Multi-omics analysis reveals *Rheum-Salvia miltiorrhiza* alleviates cisplatin-induced acute kidney injury via gut-kidney axis-mediated MAPK signaling pathway

**DOI:** 10.3389/fphar.2026.1785415

**Published:** 2026-04-15

**Authors:** Yang Zhang, Siyu Liu, Xiaoqi Li, Yuxin Zhou, Xinyan Wu, Haolan Yang, Mengjie Che, Xin Lei, Iram Laghari, Mingyue Wu, Ruilin Han, Haifeng Liu, Ziyao Zhou, Guangneng Peng, Kun Zhang, Zhijun Zhong

**Affiliations:** 1 College of Veterinary Medicine, Sichuan Agricultural University, Chengdu, China; 2 College of Food Science and Nutritional Engineering, China Agricultural University, Beijing, China

**Keywords:** acute kidney injury, gut–kidney axis, MAPK, multi-omics, Rheum–Salvia miltiorrhiza

## Abstract

**Background:**

Acute Kidney Injury (AKI) is a critical clinical syndrome with high morbidity and mortality, yet effective therapeutic agents are lacking. The *Rheum-Salvia miltiorrhiza* (R-S) combination, a traditional Chinese herbal pair, has been used to treat acute kidney injury, but its mechanisms remain unclear.

**Objective:**

This study aimed to evaluate the nephroprotective effects of the R-S combination on cisplatin-induced AKI and to elucidate its underlying mechanisms through integrated multi-omics analyses.

**Methods:**

Male C57BL/6 mice were randomly divided into five groups: Control group (Control), AKI model group (Model), *Rheum*-*S. miltiorrhiza* low-dose group (R-S-low), *Rheum*-*S. miltiorrhiza* high-dose group (R-S-high), and curcumin group (Cur). AKI was induced by a single intraperitoneal injection of cisplatin (15 mg/kg). After the experiment, renal function was assessed by measuring serum creatinine (Cr) and blood urea nitrogen (BUN). Inflammatory cytokines and oxidative stress markers were detected using ELISA. Histopathological changes of the kidney tissue were evaluated by H&E staining. Gut microbiota composition, the cecal content metabolome and the renal transcriptome were further analyze. The MAPK signaling pathway in renal tissue was examined via RT-qPCR and Western blot.

**Results:**

R-S treatment significantly improved renal function, lowering Cr and BUN, and attenuated renal histopathological injury. It also reduced oxidative stress and inflammation, elevating SOD and GSH, while decreasing IL-1β and TNF-α. Gut microbiota analysis showed that R-S restored microbial diversity, suppressed Escherichia-Shigella, and promoted Lachnospiraceae_NK4A136_group. Metabolomics identified 1237 differential metabolites, with enrichment in linoleic acid metabolism. Transcriptomics revealed 3530 differentially expressed genes, primarily associated with the MAPK signaling pathway. Molecular validation confirmed that R-S downregulated the mRNA expression of IL-1β, IL-6, TNF-α, MAPK 14, MAPK 8, NFKB 1, FOS, and JUN, and suppressed the phosphorylation of p38 MAPK, JNK, and NF-κB p65.

**Conclusion:**

The R-S combination alleviates cisplatin-induced AKI by modulating the gut microbiota, regulating metabolic profiles, and suppressing the MAPK signaling axis. This study provides a holistic, multi-omics perspective on the mechanisms of R-S, supporting its potential as a therapeutic agent for AKI.

## 1 Introduction

Acute kidney injury (AKI) is a serious medical condition marked by a swift deterioration in kidney function, leading to considerable health complications and fatalities worldwide (Babroudi and Weiner, 2025). It is recognized not only as a common complication in hospitalized and critically ill patients but also as a pivotal precursor to chronic kidney disease (CKD), thereby imposing a substantial burden on healthcare systems globally (Kumar and Gupta, 2025). The diverse causes of AKI, which encompass ischemic, nephrotoxic, infectious, and inflammatory factors, underscore the intricate nature of its underlying mechanisms and the difficulties in creating effective treatment options (Ostermann et al., 2025). Although there have been improvements in supportive care and early detection, the absence of specific pharmacological treatments remains a major clinical hurdle. This scenario calls for the investigation of new compounds that could offer kidney protection and support recovery.

Traditional Chinese Medicine (TCM) is increasingly recognized for its rich array of bioactive substances that may offer protection to the kidneys (Ma et al., 2023). The *Rheum-Salvia miltiorrhiza* (R-S) combination is a clinically well-established treatment for kidney injury in traditional Chinese medicine, known for its efficacy in improving renal function and reducing serum creatinine and blood urea nitrogen levels (Wang H. et al., 2023; Yang et al., 2025). Recent pharmacological research has started to clarify the distinct roles of these herbal components, showing their capacity to reduce oxidative stress, suppress inflammation, and enhance renal blood flow (Zhang et al., 2018; Ze et al., 2016; Sun et al., 2022; Wang et al., 2022). However, the protective effects and pharmacological mechanisms of the R-S combination against acute kidney injury have not been systematically elucidated.

Techniques for integrating multi-omics provide a systematic understanding of how microbiota, metabolites, and the host interact. By simultaneously examining the structure of gut microbiota, their metabolites, and the genomic responses in kidney tissue, a detailed pathway map can be created to demonstrate the effects of interventions. This methodology is an effective means of clarifying the complex and multi-target mechanisms of traditional Chinese medicine (Su et al., 2025). The objective of this research is to explore the protective properties of the R-S combination against kidney damage induced by cisplatin. Furthermore, it aims to combine various analyses of gut microbiota, metabolomics, and transcriptomics to shed light on its mechanisms, thus laying a theoretical groundwork for the advancement of renal protective therapies.

## 2 Materials and methods

### 2.1 Materials

*Rheum* and *S. miltiorrhiza* decoction pieces were purchased from Sichuan Neo-Gripher Chinese Herbal Pieces Co., Ltd. They were authenticated as genuine by Professor Lixia Li of Sichuan Agricultural University and met the quality standards specified in the Pharmacopoeia of the People’s Republic of China (2020 edition). The medicinal parts of both *Rheum* and *S. miltiorrhiza* are dried rhizomes. The *Rheum*-*S. miltiorrhiza* extract was prepared using a water extraction method (Qu et al., 2025). The samples are stored at the Research Center of Traditional Chinese Veterinary Medicine and Chinese Herbal Medicine, Sichuan Agricultural University, with sample number 2024010. Curcumin (C_21_H_20_O_6_, content ≥ 98.0%, HPLC) was purchased from Shanghai Yuanye Bio-Technology Co., Ltd. (B20614, Shanghai, China). ELISA kits for IL-1β, IL-6, TNF-α, T-AOC, GSH, SOD, CAT, Cr, and BUN were purchased from Cheng Nuosaisi Chemical Co., Ltd. (Chengdu). p-p38 MAPK(NO.81212-2-RR), t-p38 MAPK(NO.66234-1-Ig), GAPDH(NO.60004-1-Ig), mouse secondary (NO.SA00001-1), rabbit secondary (NO.SA00001-2)antibodies were provided by Proteintech Group, Inc(Wuhan China). p-JNK(NO.340810),t-JNK(NO.R22866), p-NF-κB p65(NO.310013), t-NF-κB p65(NO.R25149) antibodies were provided by Zhengneng Biotech Co., Ltd. (Chengdu China).

### 2.2 Determination of components in R-S

UHPLC-MS/MS analysis of R-S was conducted on a Vanquish UHPLC system coupled with an Orbitrap Q Exactive HF or HF-X mass spectrometer (Thermo Fisher). Separation used a Hypersil Gold column (100 × 2.1 mm, 1.9 μm) at 0.2 mL/min with a 12-min linear gradient. Mobile phases: positive mode-0.1% FA in water (A) and methanol (B); negative mode-5 mM ammonium acetate (pH 9.0) (A) and methanol (B). Gradient: 2% B at 1.5 min, 2%–85% B at 3 min, 85%–100% B at 10 min, back to 2% B at 10.1 min, hold to 12 min. MS parameters: spray voltage 3.5 kV, capillary temp 320 °C, sheath gas 35 psi, aux gas 10 L/min, S-lens RF 60, aux gas heater 350 °C.

### 2.3 Animal model

Male C57BL/6 wild-type mice (6–8 weeks-old) were used in the experiments (Chengdu Dashuo Experimental Animal Co., Ltd.). They were housed in a laboratory environment with constant temperature and humidity (22 °C–24 °C, 55%–60% relative humidity) under a 12-h light/dark cycle. This experiment was approved by the Animal Ethics Committee of Sichuan Agricultural University, Ethics Approval Number:20241206. After 1 week of adaptive feeding, the mice were randomly divided into the following groups (n = 10 per group) using a random number table: Control group (Control), AKI model group (Model), *Rheum*-*S. miltiorrhiza* low-dose group (R-S-low), *Rheum*-*S. miltiorrhiza* high-dose group (R-S-high), and curcumin group (Cur) (Tian et al., 2025). Curcumin, a well-documented natural compound with anti-inflammatory and antioxidant properties, was used as a positive control to validate the nephroprotective effects of R-S (Shan et al., 2024).

The dosages for the experimental animals were calculated based on the human dosage specified in the Chinese Pharmacopoeia (2020 edition). The R-S-low and R-S-high groups were orally administered the *Rheum*-*S. miltiorrhiza* extract at 0.25 g/kg/d and 0.5 g/kg/d (Yan et al., 2014), respectively. The Cur group was orally administered curcumin at 0.25 g/kg/d (Shan et al., 2024). The Control and Model groups were orally administered an equivalent volume of distilled water. Administration continued for 7 consecutive days. On day 5, a single intraperitoneal injection of cisplatin (15 mg/kg) was administered to the Model, R-S-low, R-S-high, and Cur groups to establish the kidney injury model (Chen et al., 2025). The Control group received an intraperitoneal injection of an equivalent volume of physiological saline. Forty-eight hours after modeling, the mice were anesthetized by intraperitoneal injection of 1% sodium pentobarbital (10 mg/kg). Blood was taken from the retro-orbital and immediately collected. Then mice were sacrificed by cervical dislocation.kidney tissue, and cecal content were collected for subsequent experiments.

### 2.4 Renal function assessment

Serum renal function indicators, Serum Creatinine (Scr) and Blood Urea Nitrogen (BUN), were measured in each group according to the kit instructions (Deng et al., 2025).

### 2.5 Evaluation of renal inflammatory

The levels of inflammatory cytokines in kidney tissue, IL-1β, IL-6, and TNF-α, were measured in each group according to the kit instructions (Deng et al., 2025).

### 2.6 Evaluation of renal oxidative stress

The levels of oxidative stress indicators in kidney tissue, SOD, GSH, CAT, and Total T-AOC, were measured in each group according to the kit instructions (Deng et al., 2025).

### 2.7 Renal histopathological analysis

Kidney tissue samples were fixed in 4% paraformaldehyde, dehydrated, and embedded in paraffin after wax infiltration. Sections 4 μm thick were prepared, deparaffinized with xylene, rehydrated through a graded ethanol series, and subsequently stained with hematoxylin for 4 min and eosin for 1 min. Glomerular and tubular injury scores were assessed according to published criteria (Pan et al., 2023). All histopathological changes were evaluated and photographed using an Olympus CX31 optical microscope.

### 2.8 Cecal microbiota analysis

Following collection of cecal content samples from mice in the Control, Model, and R-S-high groups, 16S rRNA sequencing was performed on the Illumina MiSeq platform (NEB, United States) by Majorbio Technology Co., Ltd. (Shanghai, China) (Wang P. et al., 2023). Prior to analysis, all sequencing data were subjected to processing on the Majorbio Cloud Platform (https://cloud.majorbio.com). The specific analytical workflow is described as follows: (1) Alpha and beta diversity indices were analyzed to reflect the community composition of different groups. (2) Based on selected distance matrices, dimensionality reduction was performed using Principal Coordinates Analysis (PCoA) and Partial Least Squares-Discriminant Analysis (PLS-DA) to identify potential principal components affecting sample community composition. (3) Using the data table from the tax_summary_a folder, community composition bar plots showing the top 10% abundant genera were generated using R language tools. (4) Linear Discriminant Analysis Effect Size (LEfSe) multi-level species discriminant analysis and multiple group comparison analysis were employed to identify genera with significant differences between groups and their effect sizes. (5) Heatmaps of communities at the genus level were analyzed, and key gut microbiota regulated by the Chinese medicine combination were investigated.

### 2.9 Cecal content untargeted metabolomics analysis

Cecal contents were collected from mice in the Control, Model, and R-S-high groups for metabolomic analysis. The LC-MS/MS analysis of sample was conducted on a UHPLC-Orbitrap Exploris 240 system equipped with an ACQUITY HSS T3 column (100 mm × 2.1 mm i.d., 1.8 μm; Waters, United States) at Majorbio Bio-Pharm Technology Co. Ltd. (Shanghai, China) (Yang et al., 2024). The data analysis procedure was conducted as follows: (1) Inter-group disparities were assessed using Partial Least Squares-Discriminant Analysis (PLS-DA). (2) Subsequent analysis focused on identifying differential metabolites and evaluating their Variable Importance in Projection (VIP) scores. (3) Finally, metabolic pathways were elucidated by mapping the identified metabolites to KEGG compound databases.

### 2.10 Renal transcriptomics analysis

Transcriptomic analysis of kidney tissue was performed by Majorbio Technology Co., Ltd. (Shanghai, China). Total RNA was extracted from the kidney tissues of mice in the Control, Model, and R-S-high groups, respectively, and its quality was assessed using a Nanodrop2000. cDNA libraries were constructed for subsequent sequencing on the NovaSeq X Plus platform (Yu et al., 2026). The data analysis was as follows: (1) Gene expression heatmaps and volcano plots were generated for differentially expressed genes (DEGs). (2) KEGG pathway and GO term enrichment analyses were performed using DAVID software (https://david.ncifcrf.gov).

### 2.11 Quantitative RT–PCR

RT–qPCR was performed by referring to previous methods (Zheng et al., 2023). In brief, extract RNA from the kidney tissues of mice in the Control group, Model group, and R-S-high group respectively, the RNA was reverse transcribed into DNA using a kit (Invitrogen, Carlsbad, California, United States). The primer sequences are shown in Table 1. The PCR process was used for quantitative detection of gene expression, followed by 40 cycles at 95 °C for 15 s, 60 °C for 30 s, and 72 °C for 30 s.

**TABLE 1:** Primers used for qRT–PCR in this study.

Gene name	Forward primer(5′-3′)	Reverse primer(5′-3′)
IL-1β	TGC​CAC​CTT​TTG​ACA​GTG​ATG	AAG​GTC​CAC​GGG​AAA​GAC​AC
IL-6	TCC​TAC​CCC​AAT​TTC​CAA​TGC​T	TGG​TCT​TGG​TCC​TTA​GCC​AC
TNF-α	ATG​GCC​TCC​CTC​TCA​TCA​GT	TTT​GCT​ACG​ACG​TGG​GCT​AC
MAPK 14	TAC​GCC​AAA​AGG​ACC​TAC​C	ATT​CCT​CCA​GTG​ACC​TTG​CG
MAPK 8	CTT​CAG​AAG​CAG​AAG​CCC​CA	TGT​GCT​AAA​GGA​GAC​GGC​TG
NFKB 1	ACA​ACT​ATG​AGA​TGA​ACT​CCG​GG	GAT​AGC​AGT​GGG​CTG​TCT​CC
FOS	TGT​TCC​TGG​CAA​TAG​CGT​GT	TCA​GAC​CAC​CTC​GAC​AAT​GC
JUN	ACC​GAG​AAT​TCC​GTG​ACG​AC	TGA​AAA​GTC​GCG​GTC​ACT​CA
GAPDH	CAT​CAC​TGC​CAC​CCA​GAA​GAC​TG	ATG​CCA​GTG​AGC​TTC​CCG​TTC​AG

### 2.12 Western blotting analysis

Western blotting was performed by referring to previous methods (Wan et al., 2025). In brief, extract protein samples from the kidney tissues of mice in the Control, Model, and R-S-high groups using lysis buffer. After separating the protein sample by SDS–PAGE, it was transferred onto a PVDF membrane. 5% skim milk was sealed for 1 h, and then the PVDF membrane was incubated overnight with primary antibody at 4 °C. The primary antibodies we used include p-p38 MAPK, t-p38 MAPK, p-JNK, t-JNK, p-NF-κB p65, t-NF-κB p65, and GAPDH. After the incubation of the primary antibodies was completed, TBST was washed three times, and then the secondary antibodies were added. The chemiluminescence method was used for imaging protein bands.

### 2.13 Statistical analysis

All experimental data are presented as the mean ± standard deviation (SD). Statistical significance between groups was assessed using a specific and precise statistical software, GraphPad Prism 8.4.0 (CA, United States), by one-way ANOVA followed by Dunnett’s test or Tukey’s HSD test for *post hoc* comparisons. In this study, a P-value <0.05 was considered statistically significant and a P-value <0.01, ***P < 0.001, ****P < 0.0001 was considered highly statistically significant.

## 3 Results

### 3.1 Component analysis of R-S

**FIGURE 1:**
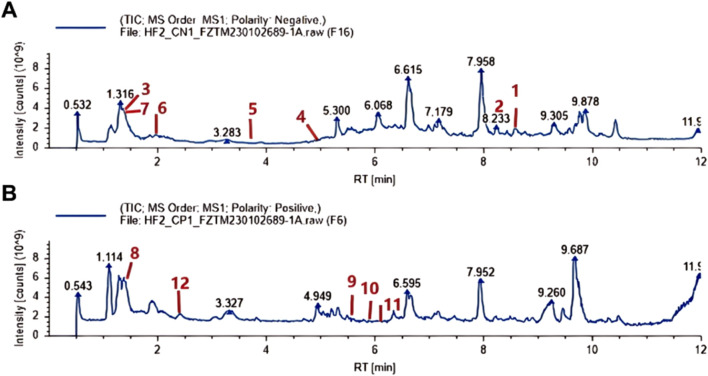
Identification of the Components of R-S using UPLC-Q-TOF-MS. **(A)** DCH sample under negative ion mode. **(B)** R-S sample under positive ion mode. (1.Anacardic acid; 2.Ginkgolic Acid; 3.Chrysophanic Acid; 4.Tanshinone I; 5.Dihydrotanshinone I; 6.Tanshinlactone; 7.Hesperetin; 8.Gallic acid; 9.Tanshinone IIA; 10.Methyl 4-hydroxy-3-methoxycinnamate; 11.Neocryptotanshinone; 12.Coumarin).

In the dual scanning mode for both positive and negative ions, R-S revealed a total of 1599 distinct chemical entities. This included 455 varieties of lipids and lipid-like substances, 164 forms of organic acids and their derivatives, 149 categories of organoheterocyclic compounds, 115 classifications of phenylpropanoids and polyketides, 82 types of benzenoids, 75 organic oxygen compounds, 52 variations of nucleosides, nucleotides, and their analogs, 22 kinds of organic nitrogen compounds, 21 types of alkaloids and their derivatives, and 11 classifications of lignans, neolignans, and similar compounds. The Total Ion Chromatogram (TIC) of R-S is shown in Figure 1; the information of the annotated characteristic components is listed in Table 2.

**TABLE 2:** Characteristic components of R-S.

Name	Formula	Molecular weight	RT [min]
Anacardic acid	C_22_H_36_O_3_	348.2662	8.583
Ginkgolic acid	C_22_H_34_o_3_	346.25083	8.203
Chrysophanic acid	C_15_H_10_O_4_	254.0574	1.402
Tanshinone I	C_18_H_12_O_3_	276.07754	4.979
Dihydrotanshinone I	C_18_H_14_O_3_	278.09328	3.724
Tanshinlactone	C_17_H_12_O_3_	264.07755	1.998
Hesperetin	C_22_ H_24_O_11_	464.13003	1.414
Gallic acid	C_7_H_6_O_5_	170.02129	1.402
Tanshinone IIA	C_19_H_18_O_3_	294.12062	5.59
Methyl 4-hydroxy-3-methoxycinnamate	C_11_H_12_O_4_	208.07339	5.919
Neocryptotanshinone	C_19_H_22_O_4_	314.15032	6.119
Coumarin	C_9_H_6_O_2_	146.03668	2.407

### 3.2 R-S ameliorated renal injury

**FIGURE 2:**
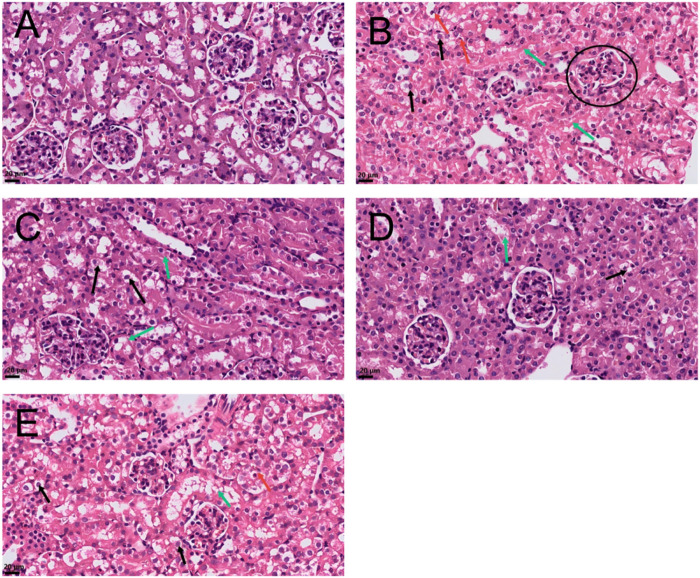
R-S alleviates renal tissue injury in AKI model mice (Scale bar represents 20 μm; image magnification ×400). **(A)** Control: no structural abnormalities. **(B)** Model: Glomerular mild congestion (circled); proximal tubular epithelial cell swelling with cytoplasmic vacuolization; nuclei displaced to one side or central in vacuoles (black arrows); epithelial cell necrosis (red arrows); abundant eosinophilic red-stained protein in tubules (green arrows). **(C)** R-S-low: Mild tubular epithelial cell swelling and degeneration (black arrows); scant filamentous protein in tubules (green arrows). **(D)** R-S-high: Proximal tubular epithelial cell swelling with cytoplasmic vacuolization; nuclei located beneath membrane or centrally (black arrows); eosinophilic red-stained protein in tubules (green arrows). **(E)** Cur: Proximal tubular epithelial cell swelling with cytoplasmic vacuolization; nuclei centrally located in vacuoles (black arrows); occasional epithelial cell necrosis (red arrows); scant filamentous protein in tubules (green arrows).

As shown in Figure 2, histopathological examination revealed that a single intraperitoneal injection of cisplatin induced acute kidney injury in mice. The Model group exhibited the most prominent lesions: extensive vacuolar degeneration of renal tubular epithelial cells, with nuclei either displaced toward the basal side or remaining centrally located. Some cells underwent necrosis and detached into the tubular lumina, accompanied by a marked increase in intraluminal protein content (Figure 2B). Following treatment, all intervention groups showed varying degrees of histological improvement. The R-S-high group demonstrated the most considerable recovery, surpassing both the R-S-low and curcumin groups (Figures 2C–E). In contrast, the Control group maintained intact renal morphology with no significant abnormalities observed (Figure 2A).

**FIGURE 3:**
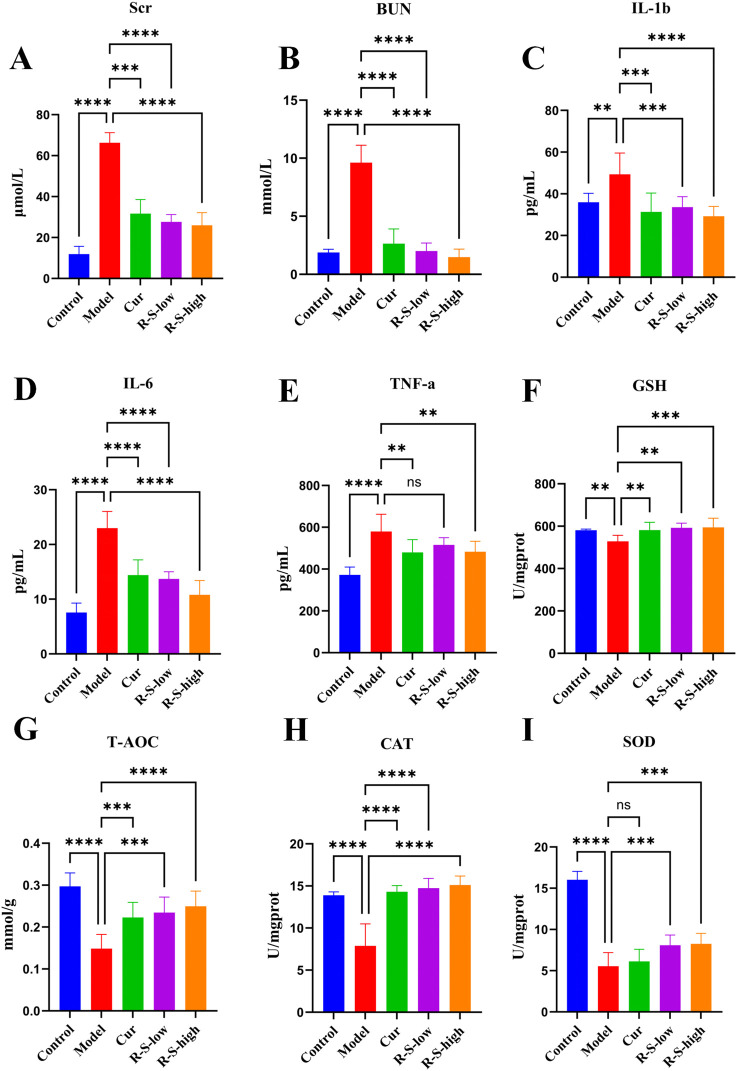
R-S ameliorates renal oxidative stress and inflammatory response in AKI model mice **(A–I)** Levels of Cr,BUN,SOD,GSH, CAT,T-AOC, IL-1β, TNF-α,IL-6. The data were expressed as the mean ± SD (n = 8), and were analyzed by one-way ANOVA. **P < 0.01, ***P < 0.001, ****P < 0.0001.

As illustrated in Figures 3A,B, the Model groups exhibited a notable rise in serum Cr levels (66.29 ± 10.99) and BUN (9.62 ± 1.50) when compared to the Control groups (***P < 0.001, ****P < 0.0001). Conversely, the R-S-low groups (Cr: 27.62 ± 8.21; BUN: 2.01 ± 0.68) and R-S-high groups (Cr: 25.95 ± 5.16; BUN: 1.48 ± 0.58) demonstrated significant reductions in these measurements relative to the Model group (***P < 0.001, ****P < 0.0001).

As shown in Figures 3C–E, compared with the Control group, IL-1β(49.39 ± 10.21), IL-6(22.98 ± 3.06) and TNF-α(579.8 ± 82.62) in kidney tissue were significantly increased in the Model group, while in the R-S-low (IL-1β,33.65 ± 5.10; IL-6:13.71 ± 1.31; TNF-α:515.6 ± 34.78) and R-S-high groups (IL-1β:29.21 ± 4.77; IL-6:10.78 ± 2.63; TNF-α:482.7 ± 50.55), those three inflammatory cytokines were significantly decreased compared to the Model group (**P < 0.01, ***P < 0.001, ****P < 0.0001).

Figures 3E–I illustrates that the Model group exhibited a notable reduction in kidney tissue levels of T-AOC (0.1486 ± 0.03), GSH (528.4 ± 28.88), SOD (5.7 ± 1.30), and CAT (7.87 ± 2.611) when compared to the Control group (**P < 0.01, ****P < 0.0001). In contrast, both the R-S-low (T-AOC: 0.23 ± 0.04; GSH: 592.5 ± 2.49; SOD: 7.85 ± 1.06; CAT: 14.75 ± 1.15) and R-S-high groups (T-AOC: 0.2496 ± 0.04; GSH: 594.3 ± 43.39; SOD: 8.02 ± 1.13; CAT: 15.11 ± 1.08) showed significant increases in these levels (**P < 0.01, ***P < 0.001, ****P < 0.0001).

Given that the R-S-high group demonstrated the most significant improvement in the aforementioned experiments, the Control group, Model group, and R-S-high group were selected for further investigation into the renal protective mechanism of R-S using multi-omics technology.

### 3.3 R-S modulated cecal microbial composition

**FIGURE 4:**
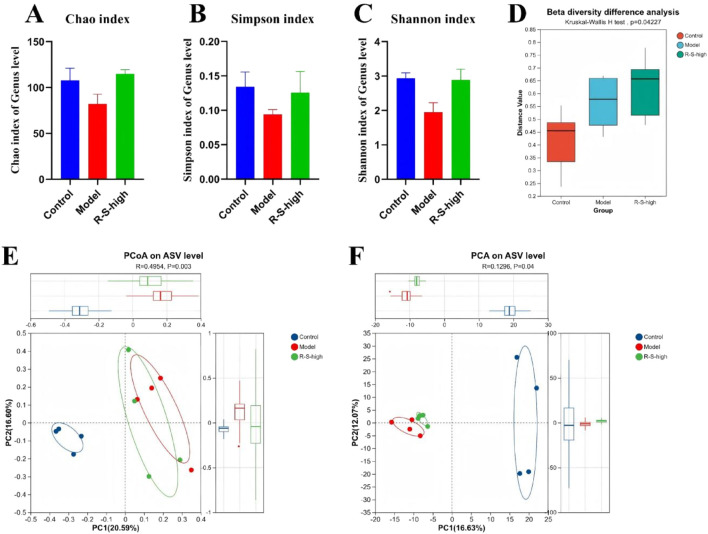
R-S modulated gut microbial diversity in AKI model mice **(A)** Chao index; **(B)** Simpson index; **(C)** Shannon index; **(D)** PCoA analyses; **(E)** PCA analyses; **(F)** Beta diversity difference analysis.

The results showed that Compared with the control group, the Shannon index (1.95 ± 0.27), Chao index (82 ± 10.71), and Simpson index (0.1 ± 0.01) of the intestinal flora in the Model group mice decreased, and through R-S intervention, the above indices (Shannon: 2.89 ± 0.30; Chao:114.8 ± 4.717; Simpson:0.13 ± 0.03) increased (Figures 4A–C).

PCoA and PCA analyses showed small within-group variations but significant between-group differences. The gut microbiota composition of the Model group and the R-S-high group were more similar, while the Control group differed significantly from both the Model and R-S-high groups (Figures 4D,E).

**FIGURE 5:**
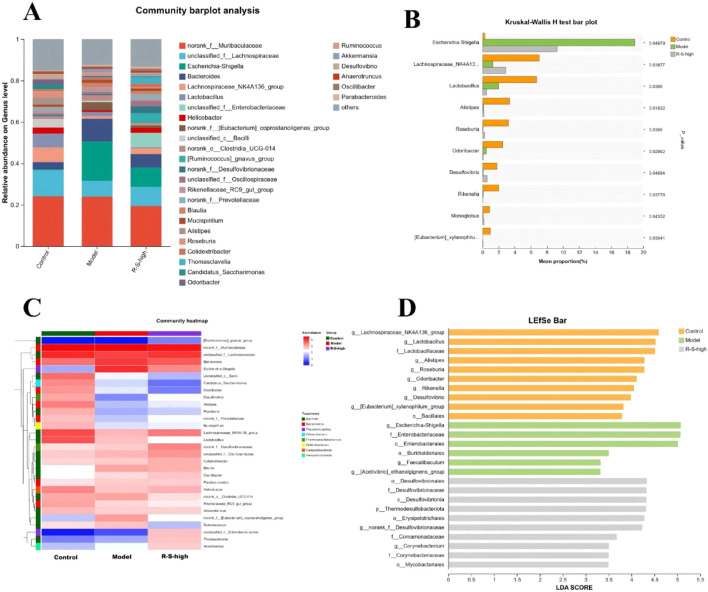
R-S modulated gut microbial structure in AKI model mice **(A)** Community composition analysis; **(B)** Differential species analysis; **(C)** Community composition heatmap **(D)** LDA score.

To determine the effect of R-S on the composition and structure of the mouse cecal microbiota, the relative abundance of dominant genera was investigated at the genus level. The top 10% genera across groups included *Muribaculaceae, Lachnospiraceae, Lachnospiraceae_NK4A136_group, Lactobacillus, Bacilli, Bacteroides, Alistipes, Roseburia, Helicobacter,* and *Odoribacter* (Figure 5A). Linear Discriminant Analysis (LDA) combined with LEfSe was used to assess the effect differences of each genus’s abundance. As shown in Figure 5D, There were 10 bacterial differential genera in group Control, 7 bacterial differential genera in group Model, and 10 bacterial differential genera in group R-S-high. Figure 5C shows the distribution of the top 30 abundant genera across groups. As shown in Figure 5B, the differential species analysis revealed that compared to the Control group, the abundance of *Escherichia-Shigella* in the Model group increased significantly (from 0.3% to 18.92%), while the abundance of *Lachnospiraceae_NK4A136_group* decreased markedly (from 7.04% to 1.25%). This trend was reversed in the R-S-high group (*Escherichia-Shigella*: 9.24%; *Lachnospiraceae_NK4A136_group*: 2.89%).

### 3.4 R-S modulated cecal content metabolism

**FIGURE 6:**
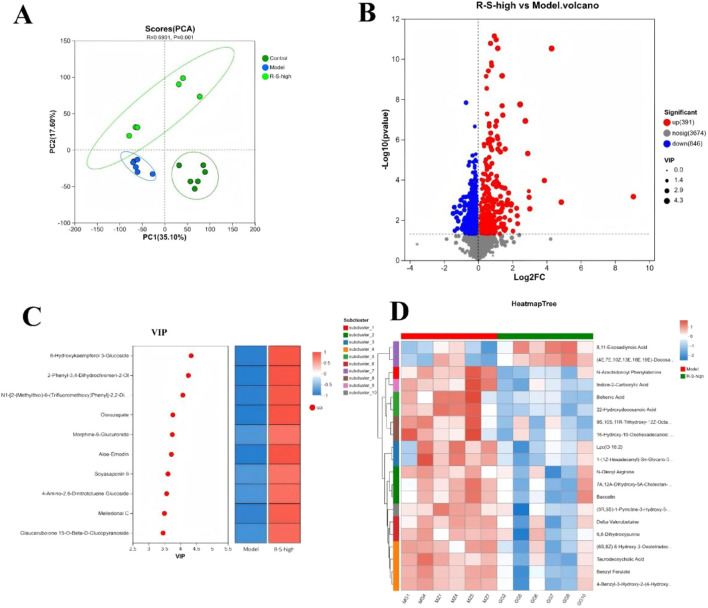
R-S modulated cecal microbiota metabolism in AKI model mice **(A)** PCA Analysis; **(B)** Volcano plot of differential metabolites in R-S-high and Model groups; **(C)** VIP analysis; **(D)** Clustering analysis of differential metabolites.

The PCA analysis revealed notable alterations in metabolic profiles across various groups (Figure 6A). The Control, Model, and R-S-high groups were distinctly separated from one another, while samples within each group displayed considerable clustering. Based on the OPLS-DA model, with VIP >1 and p-value <0.05 as screening criteria for candidate differential metabolites, a total of 1237 differential metabolites were identified between the R-S-high and Model groups. The overall distribution is shown in the volcano plot (Figure 6B). Compared to the Model group, there were 391 upregulated metabolites and 846 downregulated metabolites in the R-S-high group.

The upregulated differential metabolites in the R-S-high group were ranked by VIP value. The heatmap on the right further verifies their high expression in the R-S-high group (red blocks) and low expression in the Model group (blue blocks) (Figure 6C). Core differential metabolites such as 6-Hydroxykaempferol 3-Glucoside, 2-Phenyl-3,4-dihydrochromen-2-ol, Aloe-Emodin, Soyasaponin Ii, Melledonal C, Glaucarubolone 15-O-Beta-D-Glucopyranoside.

Cluster analysis was performed on the differential metabolites, and a cluster heatmap was generated. Model group and R-S-high group clustered into separate branches. Subcluster analysis on the left divided the metabolites into 10 categories. Metabolites in different subclusters showed characteristic expression trends in the two groups. For example, 8,11-Eicosadynoic Acid and (4E,7E,10Z,13E,16E,19E)-Docosa-4,7,10,13,16,19-Hexaenoic Acid (Docosahexaenoic Acid, DHA) was highly expressed in the R-S-high group and low in the Model group (Figure 6D).

**FIGURE 7:**
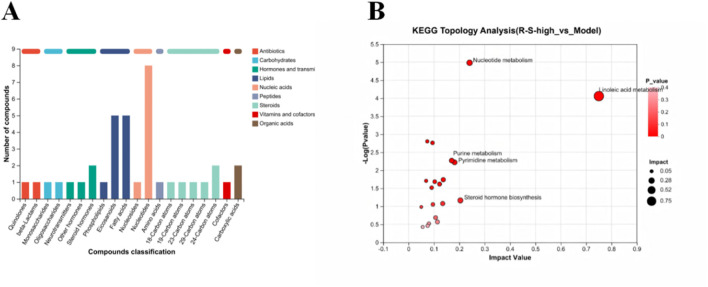
R-S modulated cecal microbiota metabolism in AKI model mice **(A)** Classification of differential metabolites by biological process; **(B)** KEGG topology analysis.

We further using biological process as the classification standard to analyze the differential metabolites according to KEGG compound categories (Figure 7A). The analysis revealed that these metabolites were predominantly found in several categories: Lipids (comprising 11 compounds: 1 phospholipid, 5 eicosanoids, and 5 fatty acids); Nucleic acids (totaling 9 compounds: 1 nucleoside and 8 nucleotides); and Steroids (including 6 compounds: 1 with 18 carbon atoms, 1 with 19 carbon atoms, 1 with 23 carbon atoms, 1 with 29 carbon atoms, and 2 with 24 carbon atoms).

To investigate the impact of differential metabolites on metabolic pathways, we performed KEGG topological analysis on the differential metabolites, and the results are shown in Figure 7B. Significant metabolic pathways identified were: metabolism of linoleic acid, nucleotide metabolism, purine/pyrimidine metabolism, and steroid hormone synthesis (Impact Value >0.15 and P-value <0.1).

### 3.5 R-S modulated renal gene transcription

**FIGURE 8:**
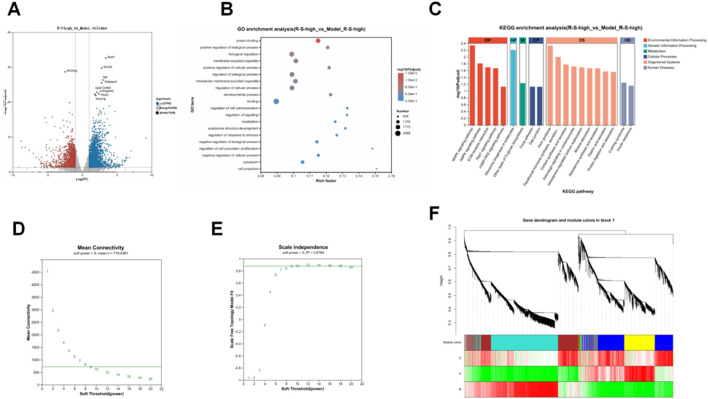
R-S modulated renal gene transcription in AKI model mice **(A)** Volcano plot of differential expressed genes in R-S-high and Model groups; **(B)** GO term enrichment analysis; **(C)** KEGG enrichment analysis; **(D)** mean connectivity curves; **(E)** scale-free topology fit index; **(F)** Gene dendrogram and module colors.

An analysis of the transcriptome showed that there were 3530 genes with differential expression between the R-S-high and Model groups, including 2182 genes that were upregulated and 1348 that were downregulated (see Figure 8A).

An analysis of Gene Ontology (GO) enrichment was conducted on the identified differentially expressed genes (DEGs). They were primarily involved in positive regulation of biological process, regulation of cell communication and signaling, anatomical structure development, and developmental process (Figure 8B). The KEGG pathway enrichment analysis of the DEGs revealed that the enriched signaling pathways mainly involved the MAPK signaling pathway, AMPK signaling pathway, ECM-receptor interaction, Rap1 signaling pathway, and cGMP-PKG signaling pathway (Figure 8C).

Weighted Gene Co-expression Network Analysis (WGCNA) was used to analyze the co-expression network in the kidney’s tissues. Figures 8D,E show the mean connectivity curves and scale-free topology fit index, respectively. Based on the scale-free topology fit index reaching a plateau, a soft threshold power of 9 was determined as optimal for constructing the co-expression network, ensuring network topology reliability. A hierarchical clustering tree (dendrogram) was then constructed based on correlation coefficients between genes. Different branches in the clustering tree represent different gene modules, and different colors represent different modules. A total of 7 modules were identified (Figure 8F).

**FIGURE 9:**
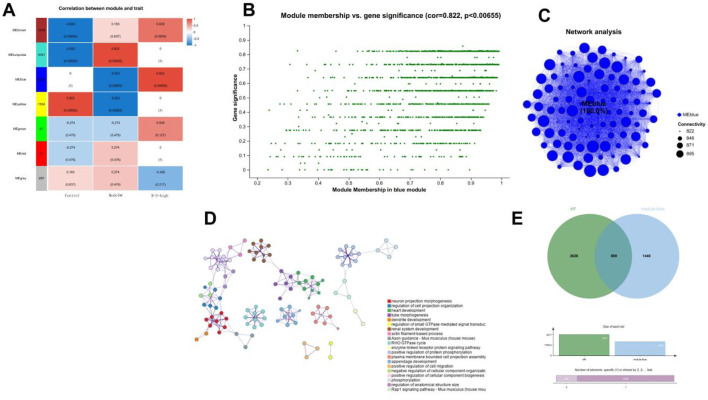
R-S modulated renal gene transcription in AKI model mice **(A)** Correlation analysis between modules and trait; **(B)** Correlation analysis between module membership and gene significance; **(C)** Network analysis; **(D)** GO enrichment analysis; **(E)** Correlation analysis between blue module genes and differentially expressed genes.

Figure 9A shows the correlation between the 7 modules and the different groups. Using a correlation coefficient |r| > 0.8 as the screening criterion, it was found that the R-S-high group had genes highly expressed in the MEblue module. Figure 9B shows the correlation between the module membership (MM) values and gene significance (GS) for genes in the blue module. The correlation coefficient (Cor) was 0.822, with a P-value less than 0.00655, indicating a significant positive correlation. An analysis of network connectivity for the MEblue module’s genes indicated a robust co-expression network (see Figure 9C). The GO enrichment analysis for these genes highlighted biological processes such as tube morphogenesis and renal system development (refer to Figure 9D). Additionally, the overlap between genes in the MEblue module and differentially expressed genes (DEGs) from the R-S-high and Model groups resulted in 889 common genes (illustrated in Figure 9E).

**FIGURE 10:**
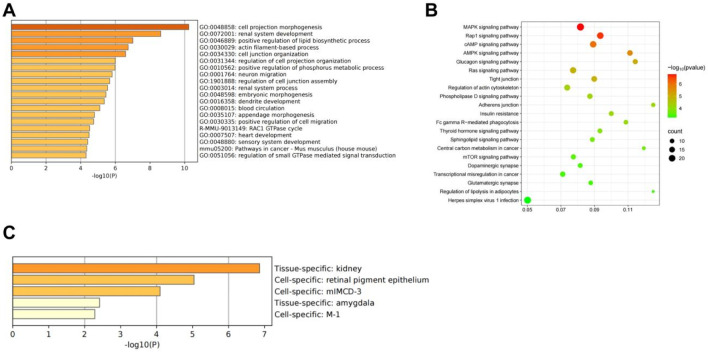
R-S Modulated Renal Gene Transcription in AKI Model Mice **(A)** GO enrichment analyses; **(B)** KEGG enrichment analyses; **(C)** Tissue-specific analysis.

We performed GO and KEGG enrichment analyses the 889 overlapping genes. The GO enrichment analysis results indicated that 889 genes were highly associated with renal system development and renal system processes. KEGG enrichment analysis indicated that the overlapping genes were highly correlated with the MAPK signaling pathway. Tissue-specificity analysis revealed that the overlapping genes exhibited kidney tissue specificity (Figures 10A–C).

### 3.6 R-S inhibited the MAPK signaling pathway in kidney tissue

Following the identification of the MAPK signaling pathway’s enrichment through transcriptomic analysis, we conducted further examinations of this pathway in kidney tissues utilizing RT-qPCR and Western blot techniques.

**FIGURE 11:**
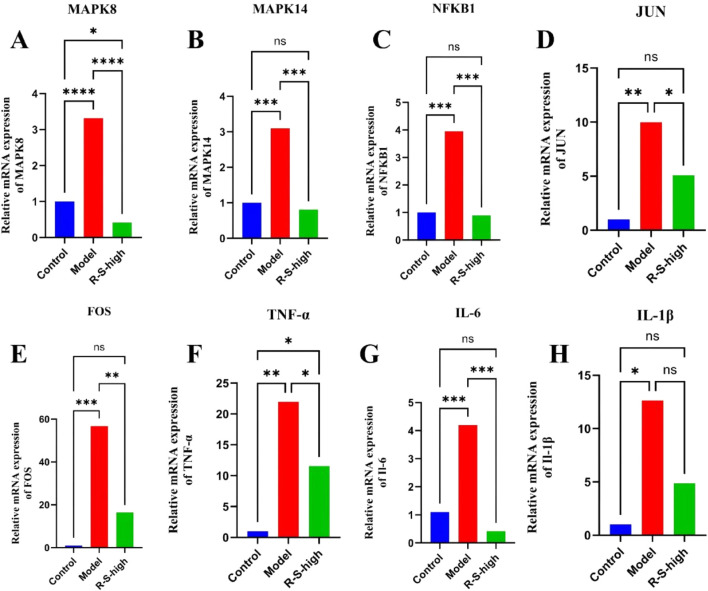
Effects of R-S on gene expression of the MAPK signaling pathway in the kidney of AKI model mice **(A)** MAPK 8; **(B)** MAPK 14; **(C)** NFKB 1; **(D)** JUN; **(E)** FOS; **(F)** TNF-α; **(G)** IL-6; **(H)** IL-1β. The data were expressed as the mean ± SD (n = 3), and were analyzed by one-way ANOVA. *P < 0.05, **P < 0.01, ***P < 0.001, ****P < 0.0001.

The findings from the RT-qPCR analysis, depicted in Figure 11, indicated that the Model group, which experienced acute kidney injury due to cisplatin, exhibited elevated mRNA levels of IL-1β, IL-6, TNF-α, MAPK 14, MAPK 8, NFKB 1, FOS, and JUN when compared to the Control group (*P < 0.05, **P < 0.01, ***P < 0.001, ****P < 0.0001). Following the R-S treatment, there was a notable reduction in the expression of these genes in the R-S-high group relative to the Model group (*P < 0.05, **P < 0.01, ***P < 0.001, ****P < 0.0001).

**FIGURE 12:**
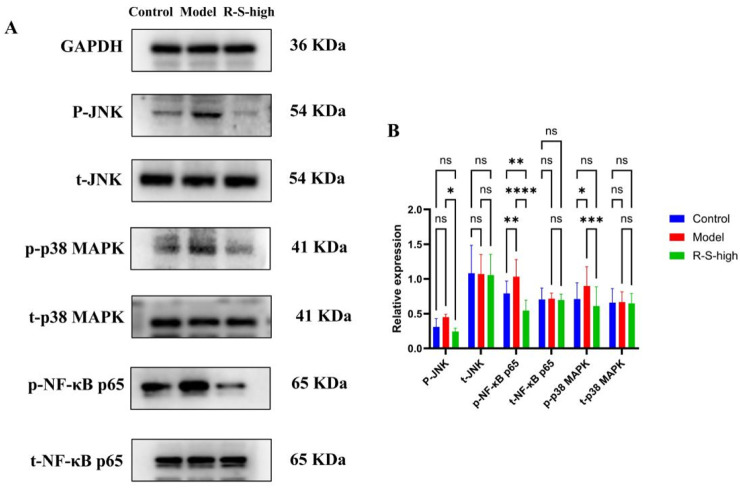
Effects of R-S on proteins expression of the MAPK signaling pathway in the kidney of AKI model mice **(A)** WB analysis of p38 MAPK, JNK, and NF-κB 65; **(B)** Comparison of the relative expression levels of p38 MAPK, JNK, and NF-κB p65 proteins. The data were expressed as the mean ± SD (n = 3), and were analyzed by one-way ANOVA. *P < 0.05, **P < 0.01, ***P < 0.001, ****P < 0.0001.

The results of the Western blot analysis, illustrated in Figure 12, indicated that the model group had higher levels of p-p38 MAPK, p-JNK, and p-NF-κB p65 compared to the Control group. Conversely, the R-S high group showed a notable reduction in the levels of these proteins when compared to the Model group(*P < 0.05, **P < 0.01, ***P < 0.001, ****P < 0.0001). Additionally, there were no significant variations in the expression of t-p38 MAPK, t-JNK, or t-NF-κB p65 across the three groups.

## 4 Discussion

AKI is a serious clinical condition characterized by rapid deterioration of renal function, yet effective pharmacological interventions remain limited (Turgut et al., 2023). Traditional Chinese Medicine offers unique advantages in treating kidney diseases through multi-target mechanisms (Zhang et al., 2025). The R-S herb combination is a well-known remedy in TCM for AKI treatment, although the precise mechanisms by which it protects the kidneys remain unclear.

The underlying pathophysiology of cisplatin-induced AKI is predominantly attributed to oxidative stress and inflammatory responses, resulting in tubular epithelial cell damage and subsequent functional decline (Xu et al., 2025). The results of our study demonstrated that R-S treatment led to a significant reduction in Cr and BUN levels, a reduction in the extent of histopathological damage, and a decrease in inflammatory cytokines (IL-1β, IL-6, TNF-α). Concurrently, there was an enhancement in antioxidant enzyme activities (SOD, GSH, CAT, T-AOC).

Kidney disease has been demonstrated to disrupt gut microbiota homeostasis, leading to dysbiosis, increased intestinal permeability, and accumulation of uremic toxins. These factors, in turn, have been shown to exacerbate renal injury (Tian et al., 2024; Qu et al., 2025; Tang et al., 2025). In the present study, the manifestation of reduced microbial diversity (Shannon, Chao, and Simpson indices) and altered microbial structure was observed in AKI mice, as indicated by an augmented abundance of the opportunistic pathogen Escherichia-Shigella and a diminished abundance of the beneficial SCFA-producing bacterium Lachnospiraceae_NK4A136_group. These findings are consistent with previous reports of gut dysbiosis in AKI (Xie et al., 2024; Si et al., 2024). R-S intervention was found to partially restore microbial diversity and reverse these dysbiotic shifts. The abundance of Escherichia-Shigella was significantly reduced, while the abundance of Lachnospiraceae_NK4A136_group was increased. It has been demonstrated that a reduction in Escherichia-Shigella contributes to the maintenance of intestinal barrier integrity and the alleviation of systemic inflammation (Duan et al., 2025). Conversely, an increase in Lachnospiraceae_NK4A136_group has been shown to promote SCFA production and reduce uremic toxin accumulation (Chen et al., 2024). These results are consistent with the mounting evidence that herbal interventions can modulate gut dysbiosis in renal diseases (Qiao et al., 2025; Lu et al., 2026).

It has been demonstrated that alterations in the composition of the gut microbiota have the capacity to reshape the host’s metabolic profile by modulating pathways such as short-chain fatty acid and amino acid metabolism (Zhang et al., 2021; Roessler et al., 2022). In order to explore the impacts of alterations in gut microbiota composition following R-S intervention, a metabolomic analysis of gut contents was conducted. The metabolomic analysis identified 1237 differential metabolites between the R-S-high and Model groups, with lipids being the category most significantly enriched. KEGG pathway analysis identified linoleic acid metabolism as the central enriched pathway. It has been demonstrated that modulation of linoleic acid metabolism has the capacity to influence key enzymes, such as lipoxygenase (LOX) and cyclooxygenase (COX), thus regulating the balance between pro- and anti-inflammatory mediators (Wang et al., 2021). In addition, a further study demonstrated that modulation of linoleic acid metabolism has the capacity to inhibit lipid peroxidation, with the products of lipid peroxidation being potent activators of the MAPK signalling pathway (Li et al., 2024). Consequently, R-S has the potential to reduce pro-inflammatory lipid mediators and peroxidation products by regulating linoleic acid metabolism, thereby attenuating upstream activating signals for the renal MAPK pathway.

In order to investigate the possibility of direct influence of these gut-derived metabolic shifts on renal inflammatory signalling, a transcriptomic analysis was performed on kidney tissues. Transcriptomic analysis revealed 3,530 differentially expressed genes (DEGs) were observed between the R-S-high and Model groups. The WGCNA analysis identified a gene module (MEblue) that exhibited a significant association with the interventional effect of R-S. Subsequent screening identified 889 core overlapping genes, which were those belonging to both the DEGs and the MEblue. KEGG enrichment analysis and tissue-specificity analysis indicated that the overlapping gene set exhibited specificity for renal tissue and was strongly associated with the MAPK signaling pathway. The MAPK pathway is a critical mediator of cellular responses to stress and inflammation. Upon being stimulated by factors such as oxidative stress, p38 and JNK proceed to phosphorylate and activate a number of transcription factors, including NF-κB and AP-1 (c-Fos/c-Jun). These in turn drive the expression of pro-inflammatory cytokines (Gülow et al., 2024; Khan et al., 2023; Fleitas et al., 2022). Consequently, R-S is hypothesised to reduce the production of inflammatory mediators and alleviate inflammatory damage by inhibiting the MAPK pathway. RT-qPCR and Western blot analyses confirmed that R-S exerts multi-layered inhibition on the MAPK pathway, as evidenced by reduced phosphorylation of p38 and JNK, downregulation of NF-κB p65 phosphorylation, and decreased mRNA expression of downstream transcription factors. The multifaceted inhibitory effects on the MAPK signalling pathway may provide a rationale for the substantial decrease in pro-inflammatory cytokines observed in this study. This mechanistic insight is further corroborated by previous literature, as several monomeric compounds identified in R-S—such as emodin, tanshinone IIA, and chrysophanol—have been independently reported to inhibit the MAPK/NF-κB axis (Xiaoming et al., 2015; Chi et al., 2023; Hu et al., 2014; Ma et al., 2024; Zhou et al., 2025; Wen et al., 2018).

Several limitations should be acknowledged. Firstly, the functional roles of the identified differential gut bacteria and metabolites, and their causal links to MAPK pathway inhibition, require experimental validation. In subsequent studies, we will seek to further substantiate these causal relationships through experimental methodologies such as fecal microbiota transplantation, metabolite supplementation, and gene knockout. Secondly, the precise molecular targets of R-S within the MAPK cascade have yet to be fully elucidated. It is important to note that these findings are based on a single preclinical model and further validation is required in diverse models and clinical settings.

## 5 Conclusion

This study utilises multi-omics approaches to elucidate the protective mechanisms of the R-S combination against AKI. R-S treatment remodelled the gut microbiota structure, altered the intestinal metabolic profile, and inhibited the MAPK signaling pathway in renal tissue. Collectively, these effects contributed to improved renal function and attenuated tissue damage. This study provides a systems-level elucidation of the nephroprotective mechanisms of this herbal pair, offering a theoretical basis for the development of R-S as a renal protective agent and highlighting the potential value of targeting the gut-kidney axis in AKI therapy.

## Data Availability

The data presented in the study are deposited in the NCBI repository, accession number PRJNA1450049 and PRJNA1450057.
